# The Rational Treatment of Common Symptoms among Infants

**Published:** 1911-04-15

**Authors:** Eric Pritchard

**Affiliations:** Senior Assistant Physician, Queen's Hospital for Children, etc.


### HOSPITAL CLINICS.

### THE RATIONAL TREATMENT OF COMMON SYMPTOMS AMONG INFANTS

By ERIC PRITCHARD, M.D., Senior Assistant Physician, Queen's Hospital for Children, etc.

(Delivered at the Polyclinic, Chenies Street, W.C., Monday February 27, 1911.)

THIS address is not a carefully argued-out treatise on any recondite subject, but merely my own experiences of the common complaints met with every day in infants. My experiences of infants in out-patients' departments and elsewhere have left me with no delusions regarding the character of most of the cases which are brought for advice. Still, I was surprised at the result of a recent analysis, for the purposes of this lecture, of a series of forty-two consecutive cases under twelve months of age which were brought to my out-patient department. In each case I asked the mother the same question—namely, what is the matter with your baby? The following represented the replies: Ten were reported to be suffering from wasting, seven from vomiting, five from fits of screaming, five from constipation, five from cough, three from diarrhœa, one from snuffles, one from retraction of the head, one from bandy legs, and the remainder from heterogeneous symptoms of an equally uninteresting character. True, some of these symptoms represented pathological conditions of some gravity and required the exercise of the brain in solving the problems. But reputations are not made by elaborate diagnoses or by the treatment of the fundamental causes of disease. What is required is relief. The commoner the disease the more important is it that we should know how to treat it properly. Since 80 per cent. of all infantile disorders are connected with food, it is clearly by attention to dietetic treatment that we are most likely to achieve success in most of the cases. This line of treatment is apparently often thought to be beneath the dignity of the specialist, whose attention seems to be centred on making an elaborate diagnosis, so that but little time is left for detailed instructions with regard to diet. Often, therefore, the mother's desire for information on this point is relieved by the presentation of a printed paper of directions for the feeding of normal infants. The use of such papers in the case of ailing infants is, in my judgment, to be deprecated; such children require individual treatment.

### WASTING.

With regard to wasting, although infants waste from general constitutional disorders, immaturity, prematurity, and specific infections, the vast majority waste because the quality of their food, the quantity, or the mode of its administration is at fault. We know that no individual, young or old, is capable of utilising for nutrition most of the articles of diet presented to him in the form of food until they have been dealt with in a special way by the processes of digestion. If proteid bodies, such as meat juice, the white of egg or lact-albumen, are injected into the circulation or into the tissues, they prove toxic, or are eliminated as foreign bodies by the kidneys. The only proteid bodies which can be utilised by the tissues for nutrition are those of a certain specific "make-up" or molecular structure. Each individual builds up his specific proteids to suit his own needs. What is true of proteid is true also of fats and carbohydrates, a fact which it is particularly important to remember with such a simple substance, for instance, as milk sugar. If this food is absorbed directly, as it sometimes is, into the circulation, it does not subserve any useful nutritional purpose, for it must be broken up into a simple mono-saccharide, that is to say, into dextrose, before it can be used.

### SOME POINTS FOR CONSIDERATION.

Nutrition cannot be furthered by forcing foreign food-substances into the system; indeed nothing short of the transfusion of the mother's blood into the infant would be of the least value from the point of view of nutrition. First the infant must learn how to propel its food onwards through the length of the alimentary canal. This is often so badly learned by the artificially-fed infant that the major part of all ingested food is returned through the cardiac end of the stomach instead of being discharged through the pyloric end into the duodenum. A consideration of the manner in which Nature teaches the newborn infant to digest human milk by the introductory apprenticeship of colostrum will show that it cannot be an easy task for it to digest cow's milk, which is difficult even for some adults. Therefore, it is courting disaster to present the newborn infant with a variety of food which so stubbornly resists cleavage into its component fragments. It will not surprise you to learn that six of the ten cases I have mentioned as suffering from wasting or marasmus were given cow's milk before they were many days old. It would be tedious to enumerate all the weaknesses or combinations of weaknesses which a critical examination of a large number of cases of wasting can disclose. But, except for some cases in which vomiting, diarrhœa, or convulsions dominate the scene, these cases can be cured with reasonable certainty by the application of a comparatively simple method of treatment.

### A TYPICAL CASE.

The type of case I mean conforms fairly well to the following description: The infant frequently vomits after feeding, the stools are too frequent, they nearly always contain so-called undigested curds, and the buttocks are red and often excoriated. The infant cries much, is restless, sleeps badly, and continuously loses weight. The history is often as follows: During the first or second day of life the midwife has administered a dose of castor oil, which has swept the bowel clear of its meconium. This is followed by a period of constipation, and you must remember that the colostrum afforded by the breast does not in any way form a basis for a motion. The colon is relatively dry and empty, and with each wave of peristalsis its unlubricated surfaces ride over one another with considerable friction, which may even cause entero-spasm. If under such circumstances an action follows, or if a second dose of castor oil has been administered in the hope of affording relief, the motion will be found to contain small white bodies which are almost always regarded as undigested casein. If these particles are washed in water, teased out, and examined they will be found to consist not of undigested curd, but strands of mucus or mucous membrane, which have been forcibly torn off the surfaces of the bowel and rolled up into little balls by the violent peristaltic movements of the intestine. If these balls are fresh they appear like raw white of egg; but if they have remained in the bowel for some hours they become opaque and look like undigested curd. All possible transitional forms are met with, from stringy glairy mucus to the opaque balls. The other day I was able to show that this was so in an infant two days old who had never had any milk of any kind; nothing but water, and then carefully prepared whey. Yet there was what appeared to be typical undigested curd in the motion. On teasing it out it was obvious it was only mucous membrane which had been torn off the bowel wall. The breaches of continuity in the mucous surface so made are a sufficient explanation of the many troubles which follow, such as the dys-peristalsis, and the extreme liability to intestinal infections, and the easy decomposition of food materials. In many such cases mothers substitute cow's milk because they feel that their own milk disagrees with the child. It is impossible to exaggerate the important part played by the meconium in lubricating the bowel and in providing a normal stool in the early days.

### Food and Feeding.

Sometimes large doses of castor oil are administered before the infant is forty-eight hours old. In all cases of marasmus I ask whether the child has had castor oil, and, if so, when. The subsequent history of infants who present these early symptoms can be easily predicted. The child is tried with first one kind of food and then another, but always with the same result, for the digestive mechanism has been dislocated from the first and no kind of food can agree. The surfaces of the colon are roughened and inefficient, both in their locomotory and absorbent functions. These children are comparatively happy when their bowels are acting freely, but uncomfortable when there is constipation. The treatment of these cases is complicated by the fact that the wasted condition of the infant invites a liberal supply of food. But very little food can be absorbed in the circumstances, and that which is not absorbed is decomposed or undergoes fermentation, and so adds to the trouble. Small quantities of food should be given, which can be easily digested and absorbed, and some indifferent material which will not decompose, but will form the basis of a motion and lubricate and soothe the irritable mucous membrane of the colon and small intestine. For hospital cases I give for an infant eight to twelve weeks old one teaspoonful of condensed milk in two or three tablespoonfuls of water every three hours, and in each feed one or two spoonfuls of petroleum emulsion. It is difficult to distinguish a meconium action from an action in a baby who has large doses of petroleum. If there is diarrhœa, I give glycerine of bismuth in the emulsion, 15 to 30 drops, according to the severity of the symptoms. The child can easily be made to take this. If there is flatulence, I give varying doses of milk of magnesia or carbonate of soda, the first of these being the more valuable. This makes a very palatable mixture. I do not think sufficient attention is given to the agreeableness of the medicines for infants. At the earliest opportunity I begin to substitute fresh milk for the condensed milk by carefully graduated increments.

### Vomiting.

Next, with regard to vomiting. Seven of the forty-two were brought for this symptom. Vomiting may appear as a symptom independently of gastric disturbance, but I shall deal only with the vomiting of gastric origin. From the point of view of treatment I classify the vomiting in reference to the time intervals after feeding at which it occurs: (1) Those where the vomiting occurs immediately after feeding; (2) where it occurs ten to thirty minutes after food; (3) where it occurs more than half an hour after the food is taken. In the first class it may be due simply to overfilling of the stomach; secondly, to the eructation of gas; thirdly, to violent movements of the stomach, increased peristaltic movements, or churning movements by the stimulating character of the food taken. In the second, it may be due to the irritable condition of the mucous membrane. In the last class it may be due to sudden coagulation of the caseinogen and the presence of a solid clot in the stomach, or to the excess of acid in the stomach. It should be noticed whether the food is returned unchanged, and whether the vomiting is unaccompanied by effort. In these cases the cause is overfilling. If the vomiting is accompanied by eructations of gas, have the child held upright, when the food will occupy the lower part of the stomach, the gas will be at the top, and can be got rid of without expelling food. Further, you should see that when the child is put to the breast it is comfortable, and that air is not sucked in between the nipple and the lips of the infant. Vomiting occurring soon after feeding and accompanied by violent movements of the stomach, which can be seen by waves of contraction passing across the abdomen, can be prevented by heroic doses of bismuth. If you give glycerine of bismuth and wait ten minutes before giving the meal, the infant is very seldom sick. Magnesia tends to correct any resulting constipation. A good prescription is:—

| | |
|---|---|
| Glycerini bismuthi carbonatis ... | ʒij. |
| Lactis magnesiæ ... ... ... | ℥ss. |
| Tinct. Auraretii ... ... ... | ♏x. |
| Aquam ad ... ... ... ... | ℥ss. |

*(To be continued.)*

